# A 3D Face Recognition Algorithm Directly Applied to Point Clouds

**DOI:** 10.3390/biomimetics10020070

**Published:** 2025-01-23

**Authors:** Xingyi You, Xiaohu Zhao

**Affiliations:** 1 National and Local Joint Engineering Laboratory of Internet Applied Technology on Mines, China University of Mining and Technology, Xuzhou 221008, China; xingyiyou@cumt.edu.cn; 2 School of Information and Control Engineering, China University of Mining and Technology, Xuzhou 221008, China

**Keywords:** 3D face recognition, deep learning, point clouds

## Abstract

Face recognition technology, despite its widespread use in various applications, still faces challenges related to occlusions, pose variations, and expression changes. Three-dimensional face recognition with depth information, particularly using point cloud-based networks, has shown effectiveness in overcoming these challenges. However, due to the limited extent of extensive 3D facial data and the non-rigid nature of facial structures, extracting distinct facial representations directly from point clouds remains challenging. To address this, our research proposes two key approaches. Firstly, we introduce a learning framework guided by a small amount of real face data based on morphable models with Gaussian processes. This system uses a novel method for generating large-scale virtual face scans, addressing the scarcity of 3D data. Secondly, we present a dual-branch network that directly extracts non-rigid facial features from point clouds, using kernel point convolution (KPConv) as its foundation. A local neighborhood adaptive feature learning module is introduced and employs context sampling technology, hierarchically downsampling feature-sensitive points critical for deep transfer and aggregation of discriminative facial features, to enhance the extraction of discriminative facial features. Notably, our training strategy combines large-scale face scanning data with 967 real face data from the FRGC v2.0 subset, demonstrating the effectiveness of guiding with a small amount of real face data. Experiments on the FRGC v2.0 dataset and the Bosphorus dataset demonstrate the effectiveness and potential of our method.

## 1. Introduction

Face recognition, including 2D and 3D recognition, has attracted a lot of interest recently because of its unique features. It is being used extensively in a variety of sectors, including affective computing, border control, criminal detection, mobile device user identification, and video surveillance [1,2,3]. Ongoing research is being carried out to develop new theories and methods with the aim of enhancing the accuracy of face recognition while ensuring its availability. Current techniques fall into two categories: deep learning-based and classic methods. Classic techniques frequently use feature extractors that were artificially created, like EigenFace [4], Fisher-Face [5], and LBP Face [6], which are well known among the well-established solutions. In contrast, deep learning techniques that naturally learn features during the training process without the need for an artificially constructed feature extractor are more popular.

Despite the success of 2D face recognition, it is still affected by occlusion, light changes, camera type, and resolution. Consequently, 3D face recognition has received more and more attention, though it is still in its infancy [3]. The problems of 3D face recognition based on deep learning are mainly reflected in the following two aspects:

(1) Deep learning methods mostly rely on data, necessitating large-scale training datasets for optimal outcomes. However, the largest available 3D face dataset currently contains only tens of thousands of training images, paling in comparison to the nearly one million images in 2D face recognition datasets like ArcFace [7], MS-Celeb-1M [8], and FaceNet [9].
(2) Developing effective network models is the foundation of deep learning methods. Existing models often operate on 2D images, neglecting the characteristics of 3D data. Point clouds, often representing 3D face data, exhibit rich geometric information and an unstructured data format. While some researchers have endeavored to employ existing deep learning networks directly on point cloud data [10,11,12,13,14,15], the efficacy of such models has primarily been demonstrated on rigid objects like chairs and tables. The uncertainties associated with face data, stemming from its unique expression and posture changes, pose significant challenges for feature extraction. This complexity underscores the need for specific deep learning network models when applied to 3D face point cloud data.

In response to the challenges encountered by 2D face recognition, such as inaccuracies due to occlusion, pose, and expression transformations, we have opted to explore 3D face recognition methods grounded in deep learning techniques. While these methods are promising, the field still has insufficient 3D face data and a lack of a network model specifically designed for 3D face data. While existing approaches have shown promise, many rely on limited training data or extensive manual preprocessing. For instance, notable methods like the one proposed by Cai et al. [16] have demonstrated significant efficacy but heavily rely on manual data preprocessing, which constrains the model’s adaptability to diverse facial structures and feature distributions, resulting in diminished performance on other datasets. Similarly, Zhang et al.’s [17] approach employs transfer learning but lacks a specifically designed network model for 3D face data, making it difficult to achieve the desired effect in real-world applications due to sample uncertainty. To address these issues, we propose a novel network model tailored to the characteristics of facial data, enabling direct processing of original 3D face point cloud data. Leveraging the distinct features of 3D face data, including varied expressions and complex postural changes, we extract 3D information representations efficiently without information loss [18,19,20,21,22,23]. To overcome data scarcity problems, we leverage synthetic methods [24], employing a Gaussian Process Morphable Model (GPMM) to generate large-scale [25], diverse face scans. With the GPMM, we create faces with random shapes, expression coefficients, and pose transformations, facilitating effective network training with minimal real-world data. Our approach, inspired by KPConv [10], presents a dual-branch network structure; these branches are tailored to handle positive neutral faces and non-neutral faces showing expression and posture changes, leveraging the advantages of dual-branch feature fusion to enhance face recognition performance. Additionally, our method incorporates a local adaptive feature learning module and employs context sampling technology to address the unique challenges posed by 3D face recognition. This approach establishes a comprehensive framework for 3D face recognition tasks, beginning with point clouds. The custom network model based on KPConv is designed to tackle the intricacies of 3D face recognition, presenting a novel solution in this evolving field.

The main contributions of this work are as follows:

(1) To diversify the training data for 3D face recognition, encompassing various identities, expressions, and poses, we introduce a data-enhanced learning framework guided by a Gaussian Process Morphable Model (GPMM). This framework enables effective network training, even with a limited amount of real data.
(2) We propose a dual-branch network structure based on KPConv, adding a local neighborhood adaptive feature learning module designed for direct facial feature extraction from point clouds.
(3) We conduct extensive experiments on established 3D face recognition benchmarks. The results show the competitiveness of our 3D face recognition method and its efficacy in addressing challenging face identification tasks in 3D space.

The rest of this paper is organized as follows:

Section 2 provides an overview of related work, while Section 3 outlines our proposed methodology. Specifically, Section 3.1 details the synthesis of a substantial volume of 3D facial scans through the GPMM in the data generator module. Our approach uses a data-augmented learning framework guided by real data, enhancing the realism of synthetic face scans. Distinguished by its efficiency in both memory and time, our method stands out among other data creation techniques. In Section 3.2, we elaborate on our strategy to utilize a KPConv-based network for extracting 3D facial representations. KPConv learns the local geometric mode of the point cloud by designing the weight of the moving kernel point, but the low-dimensional spatial coordinate relationship does not have enough ability to describe the association of adjacent points. For instance, points with the same relative position may have different semantic relations. In order to better capture facial representations, we designed a novel dual-branch network structure and added an adaptive feature learning module to replace the radius neighborhood sampling strategy of KPConv, which is used to calculate the similarity between inputs and find points and interactions between points, thereby improving the discriminability of the face recognition model in the eigenvector space and the recognition accuracy. Section 4 presents an ablation study and reports identification results on the Bosphorus [26] and FRGC v2.0 [27] datasets. The paper concludes in Section 5.

## 2. Related Work

### 2.1. On 3D Face Recognition

Li et al. [28], Guha et al. [29], and Zhang et al. [17] have contributed extensive insights into diverse 3D face recognition (3DFR) techniques. The field of 3DFR has evolved into a useful tool for facial feature identification over recent decades. These approaches are divided into two categories, classical and modern, depending on the technological procedures utilized in recognition. The classical methods focus on extracting distinct facial features—global, local, and hybrid—to facilitate matching. The global feature extraction method seeks to match all the surface features sensitive to facial expressions, such as the baseline algorithm Iterative Closest Point (ICP) introduced by Besl and McKay [30]. Yu et al., through the integration of resampling and denoising procedures into the sparse ICP algorithm, enhanced the accuracy and robustness of facial verification [31]. Local feature-based approaches, as opposed to global features, usually capture unique, compact features that reflect 3D local face information [32,33]. Guo and Da [34] focus on the investigation of a method centered on local descriptors that aims to strengthen systems’ resistance to changes in local descriptors. Hybrid feature techniques combine both global point cloud registration and local feature matching [35]. These methods represent the diversity in classical approaches for 3D face recognition, providing valuable insights into global and local feature extraction methods for matching facial features.

The deep learning approach uses complex network architectures and extensive training datasets to derive high-level, meaningful facial features from low-level information. This approach can be divided into three main types: conversion of data from three dimensions to two dimensions, advancement in network architectures, and techniques for facial reconstruction. When converting three-dimensional data to two-dimensional data, it is common to employ depth images for recognition [36,37]. Network performance has been improved by designing deep loss functions with attribute-aware loss functions, such as the one proposed by Jiang et al. [38], and incorporating face attributes like age, gender, and ethnicity into the training process. Additionally, a novel deep learning network with 3D voxel representation methods has also been used for 3D shape recognition [22], which has high memory requirements. Some approaches integrate 3D face reconstruction with deep learning, identifying features from a 3D Morphable Model (3DMM), generating dense deformable models, or locating face landmarks [39,40]. Liu et al. [41] use a cascaded regression approach to reconstruct 2D landmark location estimates along with 3D shapes. Despite their utility, these methods tend to lose 3D geometric structure information. Therefore, we design a 3D face recognition network based on point clouds using deep neural networks with 3D geometric data.

### 2.2. Deep Learning on Point Clouds

In recent years, there has been a significant surge in the application of deep learning techniques to process point clouds, addressing various challenges in this field [11,12,13,14,15]. Despite these advancements, there remains a substantial need for further exploration in the realm of deep learning on point clouds given the unique complexities associated with using deep neural networks for point cloud processing. Deep learning networks were first used for 3D point cloud processing by PointNet [11]. Using an asymmetric function, PointNet aggregated point-wise features, ensuring that they were unaffected by input point permutations. Building upon PointNet, PointNet++ [12] uses a recursive and hierarchical approach to extract both global and local characteristics from point clouds. The integration of graph CNN into point cloud processing was brought about by techniques like DGCNN [14] and SpecGCN [42]. The EdgeConv technique, which is similar to a convolution, was used in DGCNN to generate a local neighborhood graph and extract relevant local geometric information. Each layer expanded its corresponding region on the graph by considering the nearest neighbors in the feature space. Thomas et al. [10] established stiff and deformable kernel point convolution (KPConv) operators designed specifically for 3D point clouds. Without explicit optimization for facial structures, Bhople et al. [43,44] introduced identification using PointNet, which was originally designed for 3D objects (such as an airplane, chair, and desk). Obviously, it did not work out so well. Our approach builds upon the strengths of KPConv, demonstrating superior effectiveness. We devise a dual-branch network model, utilizing KPConv as the foundation, and introduce a local neighborhood adaptive feature learning module to enhance the extraction of intricate facial details, thus improving the accuracy of 3D face recognition.

### 2.3. On 3D Face Generation

The efficacy of deep learning outcomes is heavily reliant on the quality of training data. Due to the difficulty of 3D data acquisition, it is largely dependent on hardware devices, which brings challenges to the direct application of deep learning to 3D data. Existing approaches either utilize pre-existing face models or reconstruct 3D faces from images to construct training datasets [45,46,47]. For instance, Blanz and Vetter [46] enabled the generation of diverse facial models by adjusting parameters, named 3DMM, and developing a flexible and precise method to achieve fine control of face shape and appearance during synthesis. Deng et al. [45] presented a weakly supervised learning method for accurate 3D face reconstruction, while Guo et al. [47] presented an optimization technique. Gilani and Mian [48] presented a method for simultaneous interpolation across face identity and expression spaces. To address limitations in accuracy and diversity, Bhople et al. [43,44] proposed point cloud-level augmentation methods. However, these methods have constraints such as reconstruction accuracy and dense correspondence establishment. Yu et al. [49] used the GPMM method to generate training data, but they only considered shape and expression coefficients in the process of data generation and did not consider the influence of recognition pose variations. In our work, we adopt a learn-from-synthesize methodology, inspired by Yu et al. [49], aiming to automatically generate a substantial collection of annotated 3D facial scans with diverse identities. Unlike Yu et al. [49], who solely consider shape and expression coefficients in their data generation process, we extend the GPMM method by incorporating a rotation matrix during training data generation. This enhancement ensures that our training dataset encompasses not only variations in facial identity and expression but also accounts for different postural transformations, thereby facilitating effective 3D facial recognition training. As a result, our proposed 3D face recognition model demonstrates improved robustness to changes in position and posture, thereby enhancing its generalization capabilities.

## 3. Method

**Figure 1:**
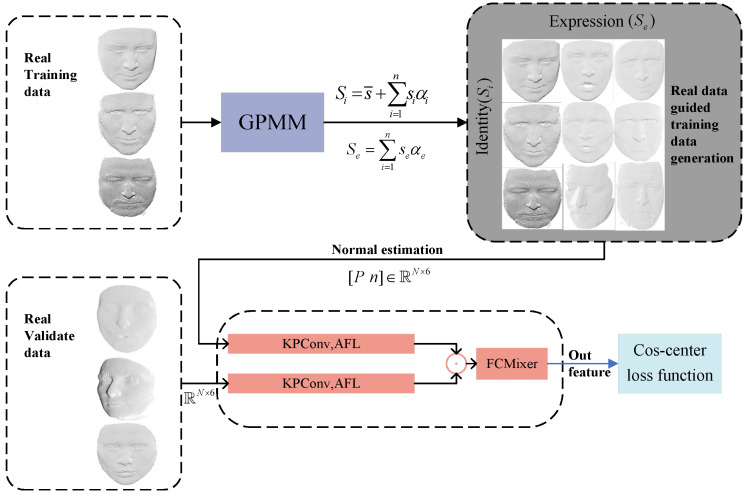
Framework for 3D face point cloud recognition.

In this section, we first prepare the synthetic data for training, then introduce our network architecture. Subsequently, we delve into the specifics of the methodology, illustrated in Figure 1, which shows the framework of the proposed approach.

### 3.1. Generation of 3D Face Data

Considering the poor performance of 3DMM’s facial details and its limited flexibility, we opted for GPMM, which offers enhanced deformation expression capabilities, to generate 3D face data. We use GPMM to learn a Gaussian distribution based on PCA, encompassing shape, texture, expression, and other attribute features, as expressed in Equation (1):

(1) $$S(p)=\bar{s}+U_{s}\left(\alpha_{s}\right)+U_{e}\left(\alpha_{e}\right)$$

where *p* represents a particular shape formed through a linear combination of the mean vector and each principal component (eigenvector). The mean of all shape vectors is represented by $\bar{s}$, where $\alpha_{s}$ is the principal component coefficient, $U_{s}$ is the eigenvector matrix, $U_{e}$ is the noise eigenvector matrix, and $\alpha_{e}$ stands for the noise coefficient.

In this research, we introduced data augmentation techniques, incorporating algorithms for facial expression alterations and face pose variations, to address challenges related to pose and noise in real-world applications. This approach aims to tackle the limitation of limited samples in existing 3D face data and enhance data diversity. Specifically, the noise in Equation (1) is modeled as facial expression changes and face pose variations, as shown in Equation (2):

(2) $$S(p)=\bar{s}+U_{s}\left(\alpha_{s}\right)+U_{e}\left(\alpha_{e}\right)+RSLG\left(\gamma \right)$$

Equation (2) introduces a noise matrix set RSLG(γ) based on Equation (1), encompassing components such as the rotation matrix *R*, scaling matrix *S*, displacement matrix *L*, and a parametric function *G* to manipulate face pose through pose parameters. As the coefficients $\alpha_{s}$, $\alpha_{e}$, and γ follow independent normal distributions, a diverse set of 3D face models is generated by randomly sampling from this distribution, incorporating various expressions and poses.

In order to bring our training data closer to real data, we introduce a strategy to generate training data using real data as the principal component coefficients. This involves calculating the expression coefficient $\alpha_{e}$ and noise parameters γ of each face in the FRGC v2.0 subset of the real-face dataset. During face generation, we randomly select $\alpha_{e}$ and γ from their respective distributions, resulting in $\alpha_{e}^{\prime }$ and $\gamma^{\prime }$.

(3) $$\alpha_{e}^{\prime }=\lambda \left(\alpha_{e}\right)-\left(1-\lambda \right)\mu_{1}$$

(4) $$\gamma^{\prime }=\lambda \left(\gamma \right)-\left(1-\lambda \right)\mu_{2}$$

where λ is (0,1), μ follows a normal distribution, and is a random value between $\mu_{1}$∼$N(0,{\sigma_{\mu_{1}}}^{2})$, $\mu_{2}$∼$N(0,{\sigma_{\mu_{2}}}^{2})$.

Subsequently, by substituting the values from Equations (3) and (4) into Equation (2), a diverse range of facial expressions resembling those found in the actual dataset can be achieved during face generation. The experimental results demonstrate a substantial improvement in the recognition rate of non-neutral faces using this approach.

**Figure 2:**
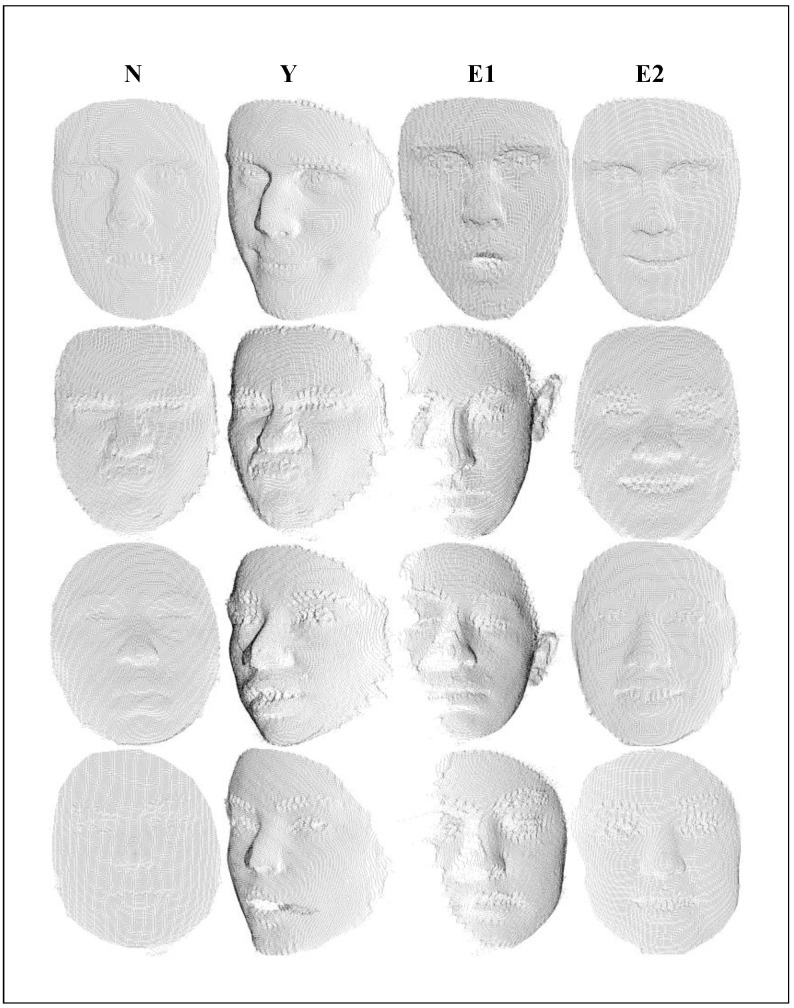
Column 1 is the face we generated, and columns 2, 3, and 4 are the noisy data after adding real-face guidance.

In the end, we generated a large-scale face dataset of 10,000 identities $\left(\alpha_{s}\right)$, each containing 200 expressions $\left(\alpha_{e}\right)$ and 120 poses (γ). Figure 2 shows the synthesized data samples, where the first column represents data without additional noise and real-face constraints, while the last three columns include real-face constraints and noise changes. Upon analysis, it is evident that our improved GPMM face synthesis data appear more authentic and reliable. Notably, in Figure 2, we can see that the facial point cloud is different from other point clouds (chairs, tables) and exhibits distinctive features concentrated in local areas such as the eyes, nose, and mouth, undergoing changes based on posture and expression variations. According to this characteristic, we propose a novel dual-branch network structure based on KPConv, featuring a local neighborhood adaptive feature learning module for the extraction of 3D facial features.

### 3.2. Network Architecture

In real-life scenarios, facial data often involve non-neutral expressions and varying poses. While existing point cloud deep learning networks exhibit strong recognition performance for neutral faces [18,48], their effectiveness diminishes when dealing with natural non-neutral faces. To address this challenge, we propose a dual-branch network structure based on KPConv. This design segregates neutral face data with robust recognition performance and non-neutral face data with natural expressions into two branches for dedicated processing. Additionally, a local neighborhood feature learning module is incorporated into one branch to selectively extract pertinent information. Subsequently, by merging the information from both branches, we achieve a more comprehensive and enriched feature representation, leading to improved accuracy in face recognition results.

The forward process of our network can be represented as follows:

(5) $$f=3DRecNet(P_{1},P_{2})$$

where $P_{i}=\left\{x_{p_{1}},x_{p_{2}},\cdots ,x_{p_{n}}\right\}\in R^{N\times 3}$ is the unordered input point cloud pair; *N* is the number of points; and $f\in R^{1024}$ is the output feature.

**Figure 3:**
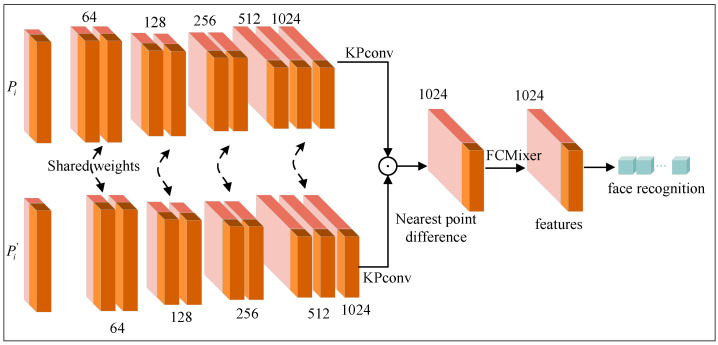
Our KPConv-based dual-branch network architecture for 3D face recognition.

Our proposed network architecture is shown in Figure 3, with subsequent subsections providing detailed introductions to each submodule.

#### 3.2.1. KPConv-Based Dual-Branch Network Structure

Our objective is to enhance the accuracy of face recognition tasks through the utilization of a dual-branch network architecture based on KPConv. This enables improved capturing of similarities across input point clouds, especially in the presence of variations in expression and posture. KPConv introduces a learnable convolution kernel, represented as a kernel point, akin to a spherical area with an adjustable radius. This kernel is employed to calculate features for each point. In Equation (6), the convolution operation is executed on the domain set surrounding each point *i*, and a weight ϖ is computed for point *j* within each neighborhood, representing the influence of point *j* on point *i*. Subsequently, the eigenvectors of the points in these neighborhoods are weighted and summed based on the corresponding weights to derive the eigenvector θ of point *i*:

(6) $$f_{\theta }\left(x_{i}\right)=\sum_{j\in N(x_{i})}\varpi (\|s_{j}-x_{i}\left\|\right)s_{j}\theta (\|s_{j}-x_{i}\left\|\right)$$

where $f_{\theta }\left(x_{i}\right)$ represents the feature of the *i*-th point, $s_{j}$ represents the position of the *j*-th point, θ is the feature vector, ϖ is the weight parameter of the convolution kernel, and $N(x_{i})$ is the neighborhood set of the *i*-th point.

**Figure 4:**
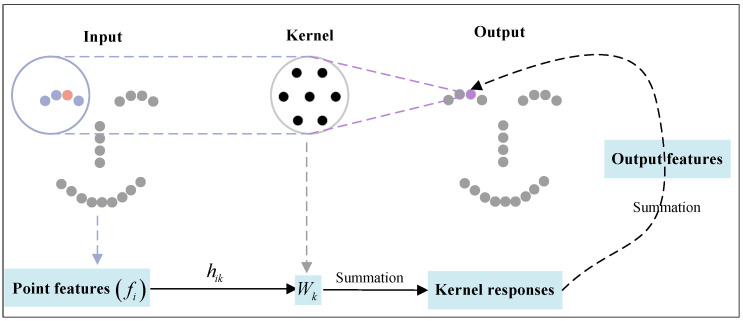
The core weight matrix $\Omega_{\theta }$ multiplies each input point feature $f_{i}$, and the correlation coefficient $h_{i\theta }$ is determined by the spatial relationship of the point with respect to the core point.

In Figure 4, each point on the point cloud is associated with a convolution kernel. Refer to Section 3.2.2 for details on the determination method of the convolution kernel.

Our dual-branch network takes a set of point clouds $\left(P_{1},P_{2}\right)$ as input and processes them through two encoders, each comprising 4 layers and 2 convolutional blocks using KPConv, as shown in Figure 3. To fuse the encoding information from the two encoders, we concatenate the feature differences corresponding to the encoding ratio. To calculate this feature difference, we designed the FCMixer function at the top of the dual-branch network. This function compares each point in $P_{2}$ with its nearest spatial point in $P_{1}$, obtaining features $\left(x_{i},x_{i}^{\prime }\right)$. A linear transformation is applied through the learnable weight matrix $\left(\omega_{1},\omega_{2}\right)$, calculating the difference between the features of each point in $P_{2}$ and the features of the nearest point in $P_{1}$. The fused feature vector $f(v_{i})$ is then obtained as a network output, indicative of the likelihood that two input faces correspond to the same individual. The decoder part of the network consists of a 4-layer stack containing recent upsampling and concatenation stages and a single convolution.

(7) $$f(v_{i})=FCMixer\left(\omega_{1}\cdot x_{i}+\omega_{2}\cdot x_{i}^{\prime }\right)$$

where $f(v_{i})$ are the fused feature vectors, $x_{i}$ and $x_{i}^{\prime }$ represent the feature vectors of the dual-branch networks, respectively, and $\omega_{1}$ and $\omega_{2}$ are the learnable weight matrices.

#### 3.2.2. Adaptive Feature Learning Module for Local Neighborhood

KPConv uses space to generate fixed convolution kernel points, computes the weight matrix through a kernel function, and processes points within a spherical neighborhood. By addressing the challenges posed by the direct and intricate regular grid convolution problem, KPConv determines the points of the spherical neighborhood through methods such as Poisson disks or random points. This approach effectively reduces operational complexity in the convolution process.

However, in 3D face recognition, traditional convolutional approaches, such as the rigid convolution kernel used in KPConv, face challenges due to the non-rigid nature of three-dimensional faces. These faces exhibit strong geometric irregularities caused by variations in expression and posture, resulting in uneven sides and fronts and clustering in specific dense areas. Unlike flat surfaces like chairs and tables, 3D faces demand a more adaptive convolutional approach. The rigid convolution kernel of KPConv, employing a system of attraction and repulsion, indiscriminately considers features from local neighborhood points, limiting its effectiveness in recognizing faces from point cloud data. For instance, attributing equal importance to the densest points in the forehead center and the regions around the eyes and nose may overlook crucial information related to facial shape and expression. Therefore, integrating contextual information from various facial areas, such as the connection between the forehead, eyebrows, and eyes, proves essential for effective 3D facial recognition.

**Figure 5:**
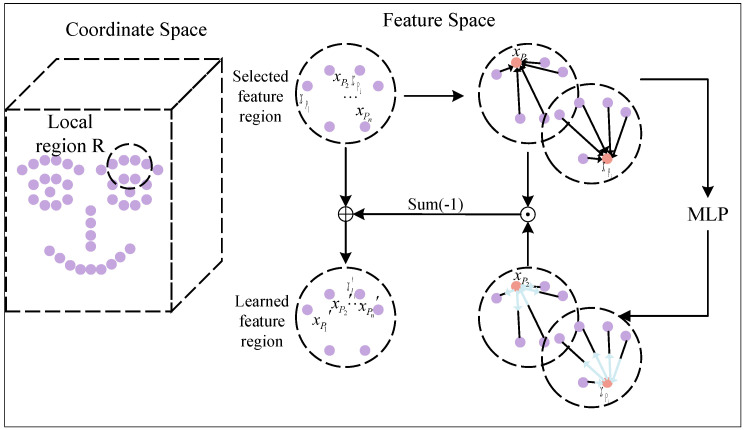
In our adaptive feature learning (AFL) module in the local region *R*, each feature $P_{i}$ experiences the influence of other features, and the strength and direction of this influence are dynamically determined by the coefficients (i) and *j* based on the differences in feature vectors. This adaptive learning process aims to enhance the descriptive power of the output $P_{i}^{\prime }$ to better capture the characteristics of the entire region.

We propose an adaptive feature learning (AFL) module designed to merge the local neighborhood features of each point in the point cloud with the contextual features of the local environment. In this process, the AFL module effectively modulates the contribution of each point to the target feature through calculating the weighted influence based on their relative positions and importance. Although traditional normalization methods are not employed during feature fusion, the adaptive calculation of each point’s influence, considering its relative relationships and significance in the local region, achieves a similar effect. This mechanism ensures balanced feature fusion while mitigating potential over-smoothing issues that can arise from conventional normalization techniques. The AFL module dynamically adjusts the radius neighborhood range, identifies neighboring points, learns the impact of each point on others to refine features, and concurrently reduces the computational burden of convolution. This adaptive adjustment enables AFL to effectively integrate inter-neighborhood contextual information into point features, significantly enhancing its capacity to characterize local neighborhoods. Figure 5 provides an overview of the AFL module, a method adept at extracting local neighborhood context through dense interconnections among points.

Given a region *R* and its feature set $P_{i}=\left\{x_{p_{1}},x_{p_{2}},\cdots ,x_{p_{n}}\right\}$, we introduce the adaptive feature learning (AFL) module. The AFL module is designed to augment point features within *P* by acquiring contextual information from local neighborhoods.

(8) $$\begin{array}{c} P_{i}^{\prime }=P_{i}+\Delta P_{i}\\ \Delta P_{i}=F\left(P_{i},P\right),\forall P_{i} \end{array}$$

where $P_{i}^{\prime }$ is the feature enhancement of $P_{i}$, and *P* is the feature set after the feature mechanism *F* is aggregated.

The feature mechanism *F* efficiently facilitates the exchange and aggregation of information within a local region *P* by adaptively learning the influence of each feature in *P* on each $P_{i}$. It is mathematically expressed as follows:

(9) $$\xi_{ij}=F(P_{i},P)=\sum_{j=1}^{n}M\left(g\left(P_{i},P_{j}\right)\right)\cdot p_{rel}\left(P_{i},P_{j}\right)$$

Here, $M(g\left(P_{i},P_{j}\right))$ computes the influence of $P_{j}$ on $P_{i}$, denoted as $\xi_{ij}$, and $p_{rel}$ represents the relationship between $P_{j}$ and $P_{i}$. Notably, we account for $P_{i}$’s self-influence on *F*.

The influence function $g(P_{i},P_{j})$ between features $P_{i}$ and $P_{j}$ is calculated using the *M* network to obtain the influence index $\xi_{ij}$ between $P_{i}$ and $P_{j}$. Here, the function *g* combines $P_{i}$ and $P_{j}$, and $\xi_{ij}$ serves as an indicator of the influence of $P_{j}$ on $P_{i}$. There are four simple ways to model a function *g*. These include no combination (AFL-non), combination by feature summation (AFL-sum), feature subtraction (AFL-sub), and feature concatenation (AFL-Con). The effectiveness of these methods is demonstrated in the following experiments. Throughout this process, the *M* network learns to calculate $\xi_{ij}$ and represent the influence of each $P_{j}$ on $P_{i}$.

The purpose of the relation function $p_{rel}$ is to ascertain how the influence indicator $\xi_{ij}$ affects $P_{i}$.

(10) $$p_{rel}\left(P_{i},P_{j}\right)=\left\{\begin{array}{cc} \;P_{i}-P_{j}, & if\;i\neq j\\ \;P_{i}, & if\;i=j \end{array}\right.$$

where when i=j, $p_{rel}$ is $P_{i}$, and when i≠j, $p_{rel}$ is $P_{i}-P_{j}$.

After the enhancement of each feature $P_{i}$ in the local region *R* by the feature mechanism, the final result is as follows:

(11) $$P_{i}^{\prime }=\alpha_{i}^{\left(i\right)}\cdot P_{i}+\sum_{j=1,j\neq i}^{n}\alpha_{j}^{\left(i\right)}\cdot \left(P_{j}-P_{i}\right)$$

(12) $$\alpha_{j}^{\left(i\right)}=\left\{\begin{array}{cc} \;-p_{imp}\left(P_{i},P_{j}\right), & if\;i\neq j\\ \;1+p_{imp}\left(P_{i},P_{j}\right), & if\;i=j \end{array}\right.$$

In accordance with Equation (12), each feature $P_{i}$ within the local neighborhood *R* undergoes the influence of a force field-like effect from the constructed adaptive feature learning (AFL) module. $P_{i}$ is subjected to forces exerted by each feature in the feature space, resulting in either attraction or repulsion. The adaptive learning coefficient $\xi_{ij}$, influenced by the difference between the two feature vectors, determines the magnitude and direction of the force. Consequently, the output $P_{i}^{\prime }$ furnishes a more comprehensive characterization of the region by incorporating contextual information from the entire region.

### 3.3. Loss Function

In addressing the significant inter-class variations and intra-class similarities observed in face data, our network optimization incorporates an enhanced central loss function. This function calculates the distance between each feature vector in the point cloud and the associated category center, utilizing cosine similarity as a replacement for the original Euclidean distance. The adoption of cosine similarity aims to assess the angle between two feature vectors, emphasizing the direction of the face data. This approach proves more effective for face learning, especially when dealing with feature vectors of varying lengths. We refer to several existing works, such as SphereFace [50] and ArcFace [7], which adopt cosine similarity to measure the similarity between facial feature vectors and achieve significant performance improvement. In addition, the application of cosine similarity has also been verified in other computer vision tasks, especially in the case of dealing with high dimensionality, sparse features, and inconsistent feature lengths. Compared with Euclidean distance, cosine similarity can provide a more stable learning process and is more effective for face learning.

(13) $$L_{c}=\frac{1}{2N}\sum_{i=1}^{N}∥d\left(x_{i},x_{i}^{\prime }\right)-c_{y_{i}}{∥}_{2}^{2}+\lambda \sum_{j=1}^{m}{∥c_{j}∥}_{2}^{2}$$

where *N* is the total number of point clouds, d(·) calculates the cosine distance between two vectors, $c_{y_{i}}$ represents the category center of the category to which the *i*-th sample belongs $y_{i}$, *m* is the total number of categories, $c_{j}$ is the category center of the *j*-th category, and λ is the regularization term coefficient.

### 3.4. Implementation Details

Trained using facial scans comprising *N* = 24,000 points, the network is utilized for classification training. Each point in the dimensional space is characterized by Euclidean coordinates (x,y,z) and associated normal vectors $\left(n_{x},n_{y},n_{z}\right)$. We train the proposed network using PyTorch. With the exception of the final classification layer, all layers are batch-normalized using the Adam optimizer. Every 20 epochs, the initial learning is lowered by a factor of 10 before being reset to $10^{-3}$. Additionally, weight decay begins at 0.5 and reduces by 0.5 each time it reaches 0.99. The network is trained on a single NVIDIA GeForce GTX 3060TI GPU for a total of 150 epochs with a batch size of 10 scans.

## 4. Experiments

First, the dataset utilized in this study and the data pretreatment processes before the experiments are described in this section. Then, for 3D face recognition, we assess the performance of the suggested dual-branch network structure using synthetic training data. Finally, we use two open-source 3D face benchmarks to evaluate our methodology.

### 4.1. Datasets

In this research, we evaluate our proposed 3D face recognition network using two publicly available datasets: FRGC v2.0 and Bosphorus.

FRGC v2.0 (Face Recognition Grand Challenge version 2.0) is a facial recognition dataset released by NIST in 2006. It contains about 1432 facial images of 466 people taken under different lighting and expressions, with individual still images, video sequences, and 3D reconstructed models, as well as gender, ethnicity, and age information. The composition of the dataset consists of two parts: Gallery and Probe. The Gallery set contains static frontal images of subjects, while the Probe set consists of query images seeking the most similar image in the Gallery, evaluating retrieval performance. In experiments, a subset of FRGC v2.0 serves as real data for network training, with 443 faces allocated for the real-data validation set and 524 faces for guiding the generation of real data.

Bosphorus, a collaborative project from Bogazici University, Turkey, focuses on collecting facial expression and shape information for 3D face modeling. It includes data from 105 individuals, with two to four facial expressions per individual, resulting in 4665 images. The dataset offers high-resolution 3D facial models with detailed information for accurate facial analysis. The Bosphorus dataset contributes to the evaluation of the proposed methodology.

### 4.2. Data Preprocessing

We conduct preprocessing on the acquired 3D face dataset obtained in Section 3.1, involving operations like point unification, normal estimation, and coordinate transformation.

The normal vector, a crucial characteristic of a point cloud, $n\in R^{N\times 3}$ is computed for the input point cloud *P* using principal component analysis. The output of the normal estimation submodule is denoted as $\left[P_{n}\right]\in R^{N\times 6}$.

In the context of 3D face recognition, the tip of the nose serves as the reference point for normalization. The coordinate transformation involves setting the coordinates of the nose tip as the origin (0,0,0) for all points in the 3D point cloud data. To mitigate potential interference from non-face areas, a sphere with a radius of 90 mm is constructed based on the nose tip. Points outside this sphere are removed, ensuring the retention of only face-related point cloud data. Additionally, potential outliers or noise are eliminated by setting a threshold for the nearest neighbor, excluding points that are excessively distant or deviate from the facial structure.

Through these preprocessing steps, non-face regions and outliers in the 3D face point cloud data are effectively addressed, providing normal estimation and a refined and consistent representation of the data with the nose tip as the coordinate origin.

### 4.3. Ablation Study

Training the network on our hardware takes approximately 80 h with a dataset comprising 5000 identities, each identity has 200 different expressions, unless otherwise specified. For the ablation investigation, the evaluation primarily focuses on the accuracy on the Bosphorus dataset, with emphasis on a subset of neutral scans identified by the filename convention “N_N_0” from the Bosphorus dataset. This approach allows for expedited and efficient comparisons.

#### 4.3.1. Effectiveness of the Local Neighborhood Feature Learning Module (AFL)

We conducted ablation studies on the Bosphorus dataset, categorizing experiments into those without the AFL module (baseline) and those with the AFL module. Following the principles outlined in Section 3.2.2 for the AFL module, we explored four different styles of the combination function g in prel within each local group. These styles included no combination (AFL-non), combination by feature summation (AFL-sum), feature subtraction (AFL-sub), and feature concatenation (AFL-Con). Quantitative results are presented in Table 1, where “rank-1 identification rate” signifies the recognition rate at which the matching item returned by the recognition algorithm ranks first among all possible matching items after comparing the query image with all items in the database.

Learning only the adjustment of individual features without interaction with other features hampers the exploitation of contextual information in local regions. Summation operations, which involve summing pairwise features, may diminish the discriminative ability for local area features, impacting recognition capability. Conversely, feature concatenation renders some feature representations nearly identical, falling short of the discriminative power achieved by the subtraction operation. The subtraction operation ensures each feature is unique to the combination of $P_{i}$ and $P_{j}$, enhancing classification ability. Consequently, we selected the local feature subtraction operation as the adjustment method due to its superior discriminative and representative characteristics compared to other alternatives. On the Bosphorus dataset, the rank-1 recognition rate increased by 1.68, demonstrating the notable effectiveness of the local neighborhood feature learning module.

**Figure 6:**
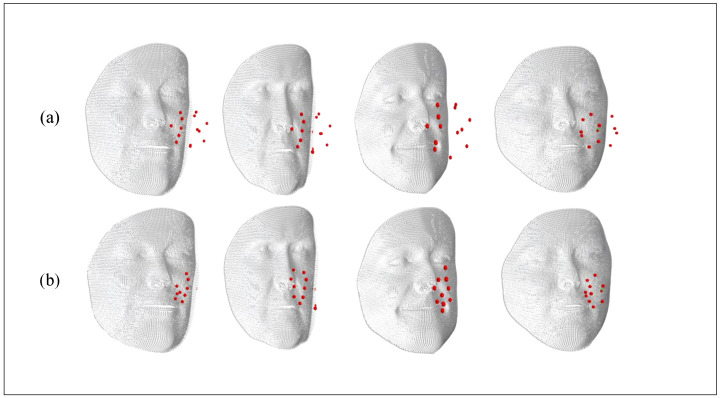
Local neighborhood feature selection, where (**a**) is without adding the AFL module and (**b**) is the change after adding the AFL module.

As shown in Figure 6, the inclusion of the local neighborhood feature learning module yields a substantial enhancement in facial feature extraction compared to scenarios where the module is absent. This improvement allows for a more effective capture of prominent feature variations among facial regions, thereby better reflecting individual differences in faces.

#### 4.3.2. Effectiveness of the Dual-Branch Network Structure

Compared with the KPConv network, the introduction of a dual-branch network structure aims to process two inputs through the same network, facilitate focused feature extraction at different scales in each branch, calculate input similarities, and share weights to reduce parameters. So that the feature advantage in positive neutral faces can be effectively applied to non-neutral faces, the subsequent merging of branch outputs achieves the fusion of information, leading to improved model training efficiency and generalization.

Table 2 outlines the configuration and training parameters of various networks, maintaining a consistent external structure for comparative experiments.

Table 3 demonstrates the consistent superiority of the dual-branch network structure, regardless of the backbone architecture (PointNet, PointNet++, and KPConv) and input form (Points, Points + Normals). Specifically, the rank-1 recognition rate of the KPConv network surpasses that of the PointNet and PointNet++ networks by 7.94 and 3.44, respectively. Moreover, the KPConv network employing the dual-branch topology achieves a higher rank-1 recognition rate (1.82) compared to the standalone KPConv network. These findings validate the significant contribution of the proposed dual-branch network and the input form using the “Points+Normals” $\left(x,y,z,n_{x},n_{y},n_{z}\right)$ layout to the improvement of 3D face recognition accuracy. Subsequent tests utilize both “Points+Normals” $\left(x,y,z,n_{x},n_{y},n_{z}\right)$ from our suggested 3D facial scans as input modalities.

#### 4.3.3. Effectiveness of the 3DRecNet Network Architecture

Here, we provide visualization results that validate the performance of the KPConv-based 3DRecNet network that uses a dual-branch network topology together with a local neighborhood feature learning module customized for 3D face recognition.

**Figure 7:**
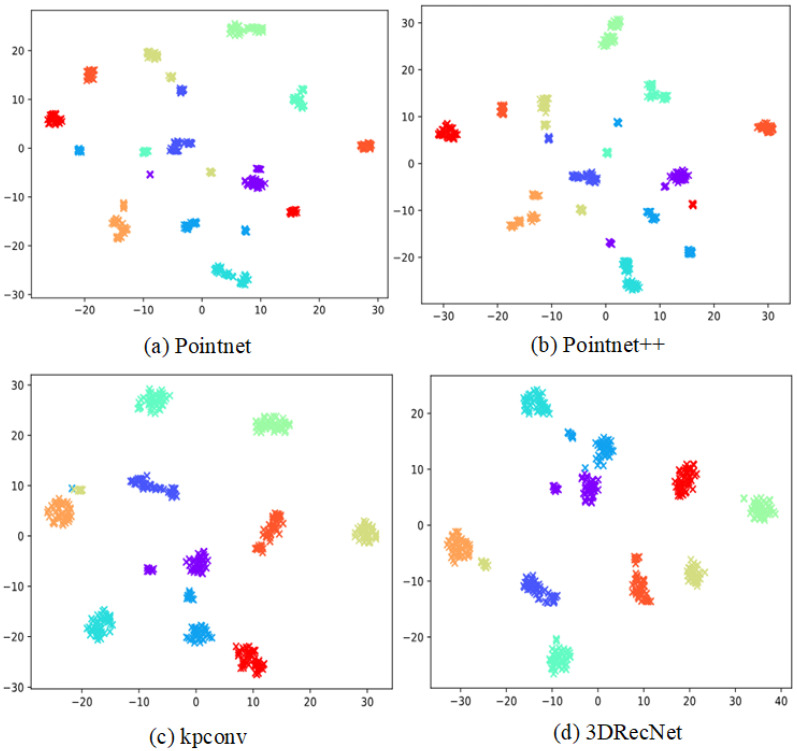
The facial features that were recovered from the different models—PointNet, PointNet++, and KPConv—as well as the suggested model, 3DRecNet, are displayed in the t-SNE visualization. Each color in the visualization corresponds to a different identity.

Figure 7 presents a t-SNE visualization of depth features obtained from various network models projected into a two-dimensional embedded space [51]. While KPConv exhibits tightly grouped depth features compared to PointNet and PointNet++, occasional clustering errors are observed. In contrast, the 3DRecNet model designed in this study demonstrates a slightly superior performance to the KPConv model, showcasing its ability to extract more discriminative features.

#### 4.3.4. Effectiveness of the Real-Data-Guided Generation

While achieving a peak accuracy of 98.83% with the initially generated dataset, our subsequent experiments revealed the potential presence of overfitting phenomena. To address this concern and validate the training efficacy, we propose a novel data generation approach guided by real data. Further experiments are conducted on constrained subsets of real data to mitigate overfitting. In these experiments, approximately half of the faces in the FRGC v2.0 sample subset are randomly selected for real-data-guided generation, forming the real-data validation set with the remaining half. The results indicate that the integration of the real-data-guided generation strategy with the initial generation method significantly enhances the rank-1 recognition rate on the Bosphorus dataset and effectively mitigates overfitting. This strategy not only sustains high accuracy but also improves the model’s generalization ability, offering a robust solution to the overfitting challenge.

**Figure 8:**
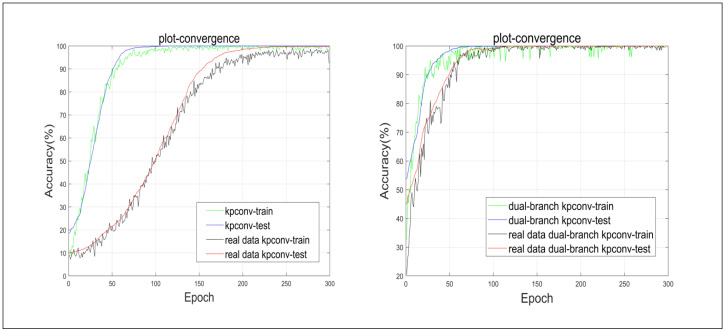
A detailed demonstration of the performance of the KPConv and dual-branch KPConv backbones is presented, considering their effectiveness on both training and test sets and whether they use real 3D face data.

Figure 8 presents accuracy curves for both the training and test sets, contrasting the baseline model with the inclusion of real data. Despite an escalation in disturbances under real-data guidance, there is a gradual rise in the accuracy rate, enhancing the generalization ability of the model. This underscores the efficacy of genuine 3D face data augmentation in addressing overfitting challenges.

#### 4.3.5. Effectiveness of the Training Data Volume

**Figure 9:**
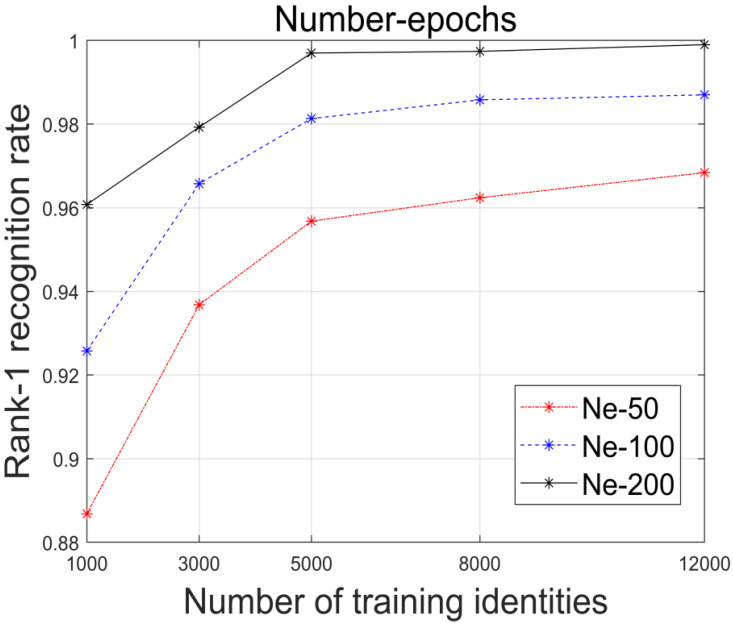
Contrasts based on various training volumes. The number of created expressions for each identity is shown by $N_{e}$.

While generating training data is needed, an excessive amount of data may lead to inefficient use of time and resources, resulting in reduced recognition efficiency. Striking a balance between recognition efficiency and the volume of training data is crucial. Through extensive experimentation, we determined that using 10,000 training samples, each comprising 200 expressions, achieved an optimal rank-1 recognition rate of 99.72% on the Bosphorus dataset, as depicted in Figure 9. Although a slightly higher recognition rate may be achievable with a larger dataset, the associated increase in time and resource costs is considered unnecessary.

### 4.4. Comparison with Other Methods

After conducting the ablation investigation in Section 4.3, we selected the 3DRecNet network architecture with the “Point+Normal” input modality as our final model. In this section, we compare our finalized model with earlier state-of-the-art techniques on two widely known public 3D face datasets: Bosphorus and FRGC v2.

#### 4.4.1. Results on FRGC v2.0

In Table 4, we present a comparison between our proposed method and other face recognition algorithms using the FRGC v2.0 dataset.

The presented table illustrates that some advancements in deep learning-based methods have demonstrated impressive recognition rates. Particularly, approaches artificial feature extraction and transfer learning, such as Zhang et al. [17] and Cai et al. [16], exhibit substantial improvements in recognition accuracy. However, the effectiveness of these methods is constrained by challenges such as the uncertainty of test samples and the need for privacy protection of sample data. Our method’s dual-branch network model designed for face-data features is still competitive among methods without transfer learning, achieving a notable 99.37% rank-1 recognition rate on the FRGC v2.0 dataset.

We evaluate the time complexity between the proposed method and the compared methods. Specifically, we analyze the computation time of preprocessing and recognition matching for each probe, since in 3D face recognition systems, the time consumption is usually due to the fact that the probe face needs to be matched with the entire gallery set. In this experiment, preprocessing includes the time to process raw 3D data and extract features. From Table 4, we can see that the time consumed by our proposed method is 2.35 s, making it the least time-consuming among all methods.

In contrast to approaches that necessitate larger datasets for marginal gains in recognition rates, our method excels in training with only the 967 faces from the FRGC v2.0 sample. Despite the limited real data, our methodology surpasses some specific approaches, underscoring its efficacy and potential.

#### 4.4.2. Results on Bosphorus

An additional experiment was carried out on the Bosphorus dataset to validate the effectiveness of our proposed method. Table 5 provides a comparative analysis of our methodology with other techniques using the Bosphorus dataset. The recognition rates are reported for an identical subset of the Bosphorus dataset to ensure a fair and consistent evaluation.

Notably, our results closely align with those of Cai et al. [16], achieving a rank-1 recognition rate of 99.72%. The key distinction lies in our method’s utilization of cosine similarity between feature embeddings as a classifier for matching scores. Furthermore, our method is nearly 0.3 percentage points higher than the recognition rate of Yu et al. [49], who also used GPMM to generate training data with a small amount of real data. This is primarily attributed to the inclusion of a rotation matrix in our GPMM method, enabling us to capture more training data reflecting changes in attitude, enhancing the generalization capability of our model. In terms of time complexity, our proposed method takes 2.06 s, second only to Zhang et al.’s [17] method, but their recognition rate is lower than ours. Therefore, compared with the existing methods, the proposed method has higher computational efficiency and can perform face recognition faster.

## 5. Conclusions

In this research, we propose 3DRecNet, an innovative end-to-end deep learning network tailored for 3D facial recognition using point clouds. To address the challenge of limited training data, our approach leverages the Gaussian Process Morphable Model (GPMM) learning-from-synthesis technique, generating diverse 3D face scans with various identities and expressions. Unlike previous methods that reconstruct 3D faces from photos or interpolate between them, our approach excels in creating realistic face scans in terms of both achieving this at larger scales and in shorter times.

Additionally, we introduce a novel point cloud network specifically designed for 3D facial recognition, addressing performance constraints in face recognition compared to generic object-based point cloud networks. Our local neighborhood adaptive feature learning module focuses on utilizing contextual cues from nearby areas to enhance face feature representation. This learning-based technique outperforms traditional 3D face recognition algorithms by capturing more abstract and high-level characteristics, providing resilience against various changes without relying on human-defined feature descriptions. In contrast to methods that incorporate depth information into 2D images to simulate or reconstruct 3D structures, our approach directly operates on point cloud data, eliminating the need for a laborious face registration phase. Employing a dual-branch network structure and various data augmentation approaches enhances training efficacy by capturing feature changes before and after processing. Comprehensive tests and comparisons on the FRGC v2.0 and Bosphorus datasets validate the superiority of our 3D face recognition system over other techniques, showcasing its resilience and efficiency in tasks such as face recognition and verification.

In future research, we aim to (1) explore face recognition with added temporal changes, including aging effects, to broaden the applicability of our method; (2) integrate generative adversarial networks (GANs) to adapt to point cloud data, address fraud concerns, and achieve improved 3D face recognition results. By combining the data diversity and realism of GAN-generated face data with the fine-grained control and robustness of the GPMM, we aim to further enhance recognition accuracy, especially in challenging conditions such as extreme facial expressions or occlusions; and (3) investigate the incorporation of meta-learning into our training framework to enhance network performance.

## Figures and Tables

**Table 1:** Whether the AFL module is added in the ablation study.

Method	Rank-1 Identification Rate (%)
Baseline	95.51
AFL-non	96.01
AFL-sum	94.37
AFL-sub	97.19
AFL-Con	96.34

**Table 2:** A list of the deep learning training parameters.

	Optimizer	Initial Learning Rate	Learning Rate Scheduler	Dropout	Loss	Batch Size
PointNet	Adam	0.001	Step	No	NLL	32
PointNet++	Adam	0.001	Step	No	NLL	24
KPConv	SGD	0.001	Exponential	Yes	NLL	10
Dual-branch KPConv	SGD	0.001	Exponential	No	NLL	10

**Table 3:** The effectiveness of dual-branch network structure on the Bosphorus database.

Method	Modality	Rank-1 Identification Rate (%)
PointNet	Points	87.53
PointNet++	Points	92.03
KPConv	Points	95.47
Dual-branch KPConv	Points	97.29
PointNet	Points+Normals	91.60
PointNet++	Points+Normals	94.10
KPConv	Points+Normals	96.75
Dual-branch KPConv	Points+Normals	98.83

**Table 4:** Rank-1 recognition rate (RR1) on FRGC v2.0 dataset.

Method	Rank-1 Identification Rate (%)	Time Cost (s)
Huang et al. [52]	97.60	3.28
Liu et al. [53]	96.94	4.4
Elaiwat et al. [54]	97.10	5.3
Lei et al. [55]	96.30	3.16
Gilani and Mian [48]	97.06	4.02
Gilani et al. [56]	98.50	3.8
Cai et al. [16]	100	3.57
Zhang et al. [17]	99.46	2.6
Yu et al. [49]	98.85	4.43
Ours	99.37	2.35

**Table 5:** Rank-1 recognition rate (RR1) on Bosphorus dataset.

Method	Rank-1 Identification Rate (%)	Time Cost (s)
Huang et al. [52]	97.00	3.16
Liu et al. [53]	95.63	4.08
Berretti et al. [57]	95.67	5.25
Lei et al. [55]	98.90	2.9
Gilani et al. [56]	98.50	3.55
Cai et al. [16]	99.75	3.3
Zhang et al. [17]	99.68	1.82
Yu et al. [49]	99.33	5.46
Ours	99.72	2.06

## Data Availability

The data presented in this study are openly available at https://www.nist.gov/programs-projects/face-recognition-grand-challenge-frgc (FRGC V2.0) accessed on 5 March 2024 and https://github.com/huyhieupham/3D-Face-Recognition?tab=readme-ov-file (Bosphorus) accessed on 10 April 2024.
